# Long term responders in frontline multiple myeloma—exception vs expectation of the modern era

**DOI:** 10.1038/s41408-024-01100-z

**Published:** 2024-07-17

**Authors:** Muhamed Baljevic, Douglas W. Sborov, Shaji K. Kumar

**Affiliations:** 1 Vanderbilt-Ingram Cancer Center, Vanderbilt University Medical Center, Nashville, TN USA; 2 Huntsman Cancer Institute, University of Utah, Salt Lake City, UT USA; 3 Mayo Clinic Comprehensive Cancer Center, Rochester, MN USA

**Keywords:** Prognosis, Cancer therapy

Described as *mollities ossium* in 1844, the first reported case of a patient with multiple myeloma (MM) was a 39 year-old-woman treated with an infusion of orange-peel, rhubarb, and opiates as needed for palliation. At the time of her presentation, it was apparent that her disease had been present for several years and her struggle lasted only 5 more days [1]. Too young to die, too futile the treatment. Fast forward nearly two centuries and following the introduction of key foundational therapeutics including immunomodulatory drugs (IMiDs), proteasome inhibitors (PIs) and autologous stem cell transplantation (ASCT), the possibility of providing patients extended survival and improved quality of life has become a reality. In the 2000s, triplet combination therapy for patients with newly diagnosed multiple myeloma (NDMM) included bortezomib, lenalidomide, and dexamethasone (VRd) followed by a risk-stratified maintenance treatment. In one such study where 75% and 17% of patients underwent upfront and deferred ASCT, respectively, a median progression-free survival (PFS) was 65 months (5.4 years) and the median overall survival (OS) was 126.6 months (10.5 years) for the entire cohort. The fact that patients with high-risk (HR) disease did markedly worse than those with standard risk (SR) disease, highlighted that while the new therapeutics were increasingly effective, there was clearly a population of patients that continued to have limited survival. The median PFS for HR and SR patients was 40.3 months (3.4 years) and 76.5 months (6.4 years) [2], respectively, and the median OS after 88.4 month of follow-up for HR and SR was 86.6 months (7.2 years) and not reached (NR), respectively [3]. An improvement for some, but clearly room for further refinement.

Relative to prior experience and standards, the notion of long-term responders (LTRs), particularly in those considered to be eligible for ASCT (TE) at the time of initial diagnosis slowly became more routine rather than the exception to the rule. That said, the addition of a CD38-directed monoclonal antibodies (daratumumab or isatuximab) to a lenalidomide-based triplet combination including either VRd or carfilzomib (KRd) has ushered in the modern era of powerful induction regimens for patients with TE NDMM. Overall response rates are nearly 100% and responses are deep for most. Minimal residual disease (MRD) negative responses are being achieved in two thirds to three quarters of treated patients by the end of consolidation and maintenance, with sustained MRD negativity observed well beyond 12 months, particularly in SR patients. As such, these new approaches lay a fertile ground for the achievement of long-term response in even greater proportion of patients. Outlined in Table 1 are some of the recent triplet and quadruplet studies in the TE NDMM population that reflect these new achievements and standards [4–12]. The latest development of chimeric antigen receptor-directed T cells (CAR-T) and bispecific antibodies (BsAbs) will continue to reshape the therapeutic landscape and may even improve outcomes in those with HR, more treatment resistant disease.

**Table 1:** Modern landscape of triple and quadruple regimens in transplant eligible newly diagnosed multiple myeloma.

Feature	PETHEMA/GEM2012 [4]	IFM-2009 [5]	DETERMINATION [6]	CASSIOPEIA [7]	GRIFFIN [8]	GMMG-HD7 [9]	PERSEUS [10]	GMMG -CONCEPT [11]	IsKia [12]
*N*	458	700	722	898	203	662	709	TE: 99	302
Phase of study	3	3	3	3	2	3	3	2	3
Treatment combination	VRd	VRd +/− ASCT	VRd +/− ASCT	R1: DVTd vs VTd R2: Dara vs placebo	DVRd vs VRd	IsaVRd vs VRd	DVRd v VRd	IsaKRd	IsaKRd vs KRd
Primary endpoint	PFS	PFS	PFS	R1: sCR p consolidation R2: PFS p maintenance	sCR p consolidation	R1: MRD- p induction R2: PFS p maintenance	PFS	MRD (-)10^−5^	MRD (-)10^−5^
Induction/consolidation	6/2 (28-day)	3/2 (28-day)	3/2 (21-day)	4/2 (28-day)	4/2 (21-day)	3/0 (42-day)	4/2 (28-day)	TE: 6/4 (28-day)	4/4 (28-day)
Maintenance duration	NR	Len 1 year	Indefinite	2 years	2 years	3 years	Indefinite	Indefinite	NR
Median follow-up (mo)	NR	90	76	45	50	NR	48	TE: 44	20
ORR (%)	80.6	NR	98 vs 95	92 vs 90	99 vs 92	90 vs 84	97 vs 94	TE: 95	NR
≥VGPR (%)	76	78 vs 69	83 vs 80	83 vs 78	96 vs 77	77 vs 61	95 vs 89	TE: 91	94 both
Post-consolidation sCR (%)	50 (CR)	NR	33 vs 28	29 vs 20	42 vs 32		69 vs 45	TE: 73	64 vs 67
MRD(−) 10^−4^(%)	66	79 vs 65	NR	NR	NR	NR	NR	NR	NR
MRD(−) 10^−5^ (%)	NR	NR	55 vs 40	NR	64 vs 30	NR	75 vs 48	NR	77 vs 67
MRD(−) 10^−6^ (%)	NR	30 vs 20	NR	NR	36 vs 16	NR	65 vs 32	NR	67 vs 48
P-induction 10^−5^ (%)	29 (10^−6^)	NR	NR	NR	22 vs 8	50 vs 36	NR	NR	NR
P-ASCT 10^−5^ (%)	42 (10^−6^)	NR	NR	NR	NR	NR	NR	NR	NR
P-consolidation 10^−5^ (%)	66 (10^−4^)	NR	NR	57 vs 37	50 vs 20	NR	NR	68 vs 54	77 vs 67
P-maintenance 10^−5^ (%)	NR	NR	NR	NR	64 vs 30	NR	NR	82 vs 69	NR
MRD(−) 10^−5^ ≥ 12 mo	NR	NR	NR	NR	NR	NR	65 vs 30	63 vs 46	NR
4-year PFS (%)	NR	NR	NR	NR	87 vs 70	NR	84 vs 68	NR	NR
Median PFS (mo)	NR	47 vs 35	68 vs 46	NR vs 47	NR vs NR		NR	NR	
OS (%)	NR	62 vs 60 (8-year)	81 vs 79 (5-year)	NR	NR	NR	NR	NR	NR

*N* number, *TE* transplant eligible, *VRd* bortezomib, lenalidomide, dexamethasone, *ASCT* autologous stem cell transplant, *DVTd* daratumumab, bortezomib, thalidomide, dexamethasone, *VTd* bortezomib, thalidomide, dexamethasone, *R1* first randomization, *R2* second randomization, *Dara* daratumumab, *DVRd* daratumumab, bortezomib, lenalidomide, dexamethasone, *IsaVRd* isatuximab, bortezomib, lenalidomide, dexamethasone, *IsaKRd* isatuximab, carfilzomib, lenalidomide, dexamethasone, *KRd* carfilzomib, lenalidomide, dexamethasone, *PFS* progression-free survival, *sCR* stringent complete response, *p* post, *mo* month, *MRD* minimal residual disease, *Len* lenalidomide, *NR* not reported, *ORR* overall response rate, *VGPR* very good partial response, *OS* overall survival.

In this issue of *Blood Cancer Journal*, Pasvolsky and colleagues sought to describe characteristics of LTR patients who achieved a prolonged PFS (≥8 years) following upfront ASCT and identify predictors of favorable outcome [13]. In the largest cohort of such analysis to date (*n* = 1576), LTRs accounted for 15% of the patients. At a median follow-up of 126.1 months (10.5 years) for the LTR cohort, the median PFS was 169.3 months (14.1 years) vs 26.5 months (2.2 years) in the non-LTR group, with the median OS NR vs 81.3 months (6.8 years), respectively [13]. Compared to the control non-LTR group, LTR patients were: (1) younger (median age 58.4 vs. 59.5 years; *p* = 0.012), (2) harbored HR cytogenetics less frequently (4% vs. 14%; *p* < 0.001), (3) had R-ISS stage I disease more commonly (43% vs. 34%; *p* = 0.010), (4) had lower disease burden (<50% bone marrow plasma cells at diagnosis) (67% vs. 58%; *p* = 0.018), (5) more often achieved ≥VGPR (50% vs. 41%; *p* = 0.009) and MRD negativity prior to ASCT (52% vs. 35%; *p* = 0.016), (6) had higher rates of CR at day+100 post-ASCT (41% vs. 27%; *p* < 0.001) and as best post-ASCT response (70% vs. 37%; *p* < 0.001), and (7) more often received post-ASCT maintenance (63% vs. 52%; *p* = 0.002) [13]. Important to note, the exceptional results from the present study stem from an analysis done in the era (2000–2014) when quadruplet inductions followed by doublet-maintenance approaches was not a standard in TE NDMM. In fact, the most common induction therapy for these LTRs was a doublet of IMiD + dexamethasone (29%) followed by VRd (18%), while no patients were treated with CD38-directed monoclonal antibody-containing regimens or quadruplet induction therapy. No maintenance treatment was given to 37% of LTRs, which is comparable to other reported studies (Table 4) [13].

Nevertheless, extraordinary LTRs seems to represent a small proportion (~10–15%) of MM patients who despite their superior early disease control still require life-long care, reaffirming that despite major strides in treatment options, MM remains incurable for the majority. In the current study, permanent durability of response among LTRs was not implied by the PFS Kaplan–Meier curve (i.e., no plateau) and the leading causes of death were still disease progression (35%) and second primary malignancies (SPMs) (22%) [13]. It always seems easiest to focus on these patients with exceptional responses, however, most patients continue to face the reality of not living to their expected age. What remains clear is that while options continue to improve, we still have work to do.

Fixed-duration MRD-guided approaches represent a new wave of exploration in early MM course for all comers as we continue to observe that some patients are overtreated while others are undertreated. Some of these next generation trials are reconsidering the role of ASCT, especially given the question of OS benefit, and examining the comparative impact of CAR-T and T-cell redirecting BsAbs. Beyond the desire to test the additive impact of early adoption of these powerful agents in the front-line setting, some hope to avoid the concerns of mutagenesis of hematopoietic pool caused by high dose melphalan, as a potential important factor associated with the development of SPMs among MM patients who undergo ASCT [14]. In general, developing evidence based approaches to deescalate therapy in the appropriate settings of adequately sustained MRD-negativity, as well as learning at what point one needs to escalate treatment, to what extent, and in what form are the focus points of current and up-and-coming randomized clinical trial designs that hope to mature the modern era of MRD-informed MM action plans. While the rational treatment de-escalations can bring about reduction in adverse event profiles, lower rate of undesirable long-term events such as SPMs, and ease the burden of treatment-associated financial toxicity to patients and their families, this needs to be coupled with appropriate personalized monitoring plans to guide optimal and timely treatment decisions. Lastly, as we strive to leverage the nature of the MM amenable to prolonged responses observed across various LTR cohorts, we are appreciating the fact that immune competence, effector-cell fitness and disease and treatment related dysfunction and/or suppressions of optimal immune surveillance likely play a very important role [15]. Greater awareness of immunologic competency is therefore an essential component of maximizing therapeutic impact of all modern anti-MM therapies.

Deeper understanding of LTRs represents a valuable step in our understanding of MM landscape. As we strive towards therapeutic solutions which may allow prolonged treatment-free intervals in this disease, LTR patients with their patient and disease-related characteristics likely represent the most optimal target for the achievement of an elusive goal of cure, in at least subset of patients with MM. Availability of novel therapies (e.g., CAR T-cell, BsAbs) and their early incorporation may hopefully significantly increase the LTR fraction in years to come.
